# A Survey of Emergency Medicine Residents’ Use of Educational Podcasts

**DOI:** 10.5811/westjem.2016.12.32850

**Published:** 2017-01-30

**Authors:** Jeff Riddell, Anand Swaminathan, Monica Lee, Abdiwahab Mohamed, Rob Rogers, Salim R. Rezaie

**Affiliations:** * University of Washington, Department of Medicine, Division of Emergency Medicine, Seattle, Washington; † New York University, Department of Emergency Medicine, New York, New York; ‡ University of Texas Health Science Center at San Antonio, Department of Emergency Medicine, San Antonio, Texas; § University of Kentucky, Department of Emergency Medicine, Lexington, Kentucky; ¶ Greater San Antonio Emergency Physicians, San Antonio, Texas

## Abstract

**Introduction:**

Emergency medicine (EM) educational podcasts have become increasingly popular. Residents spend a greater percentage of their time listening to podcasts than they do using other educational materials. Despite this popularity, research into podcasting in the EM context is sparse. We aimed to determine EM residents’ consumption habits, optimal podcast preferences, and motivation for listening to EM podcasts.

**Methods:**

We created a survey and emailed it to EM residents at all levels of training at 12 residencies across the United States from September 2015 to June 2016. In addition to demographics, the 20-question voluntary survey asked questions exploring three domains: habits, attention, and motivation. We used descriptive statistics to analyze results.

**Results:**

Of the 605 residents invited to participate, 356 (n= 60.3%) completed the survey. The vast majority listen to podcasts at least once a month (88.8%). Two podcasts were the most popular by a wide margin, with 77.8% and 62.1% regularly listening to *Emergency Medicine: Reviews and Perspectives* (*EM:RAP*) and the *EMCrit Podcast*, respectively; 84.6% reported the ideal length of a podcast was less than 30 minutes. Residents reported their motivation to listen to EM podcasts was to “Keep up with current literature” (88.5%) and “Learn EM core content” (70.2%). Of those responding, 72.2% said podcasts change their clinical practice either “somewhat” or “very much.”

**Conclusion:**

The results of this survey study suggest most residents listen to podcasts at least once a month, prefer podcasts less than 30 minutes in length, have several motivations for choosing podcasts, and report that podcasts change their clinical practice.

## INTRODUCTION

A podcast is a digitally recorded media product that can be downloaded or streamed, typically as an audio file.^1^ Emergency medicine (EM) educational podcasts have become increasingly popular for learning and are one of the most widely consumed digital educational tools.^2^ Their exponential growth is evidenced by over 15 million downloads of the *EMCrit* (Emergency Medicine Critical Care) *Podcast* and the more than 24,000 paid subscribers to *EM:RAP* (Emergency Medicine: Reviews and Perspectives).^3^,^4^

A recent survey on asynchronous learning among United States (U.S.) EM residents showed that residents spend a greater percentage of their time listening to podcasts than they do using other educational materials, including textbooks and journals. They also rated podcasts as the most beneficial use of their time.^5^ A similar survey of Canadian physicians found that 90% of EM residents used podcasts every month. Despite their popularity, little is known about this phenomenon, which has led EM educators to call for a deeper understanding of how and why learners use podcasts.^6^

While there has been a dramatic recent increase in the number of EM educational podcasts,^2^,^7^ research into podcasting in the EM context is sparse. Though educators are now beginning to define quality indicators in EM podcasts,^8^ little is known about motivation, adoption, usage patterns, or preferences in consumption among podcast listeners.^9^ Outside of EM, small survey studies exist in undergraduate medical education,^10^ anesthesia,^11^ and nursing training,^12^ yet the existing literature does not provide insight into the unique EM educational landscape.

As we adopt new technologies, we must also understand how and why they are being embraced by our learners in order to employ them more effectively. The important questions of why residents are using podcasts and how they are being used remain unanswered.^13^ The personal, social, and technological factors that influence the use of EM podcasts – factors known to influence learning – merit further exploration.^14^

We aimed to better understand factors driving the asynchronous podcast phenomenon, including consumption habits, optimal podcast preferences, and motivation for listening.

## METHODS

### Study Design and population

This study was performed between September 2015 and June 2016. It was approved by the institutional review board at the University of Texas San Antonio.

We followed accepted guidelines for survey development in medical education research.^15^ We created an electronic survey via Google Forms (Mountain View, CA) and sent a link to it in a solicitation email to EM residents in all levels of training at a sample of 12 EM training programs (n = 605). Based on an estimated population size of approximately 6,000 with a 5% margin of error, we estimated we needed approximately 360 respondents to reach a 95% confidence level. Due to historically low survey response rates in multi-institutional studies of health professions trainees^16^ and the fact that recognition and trustworthiness of the survey sender may increase response rates,^17^ we used a network strategy for program selection and survey implementation. We chose residencies to represent a geographical spread across the U.S. with a mix of public, private, military, three- and four-year programs, rural, and urban environments that had a local program director personally known to the authors. Either an author or faculty member at each residency sent the email with the survey, as well as reminder emails (up to six). All responses were anonymous. All programs were approved by the Accreditation Council for Graduate Medical Education.

### Validity Evidence for Survey Items

After a thorough literature review, interviews with residents and faculty at two institutions (UW and UCSF-Fresno) and synthesis of background information, we developed questions. Several authors (AS, RR, SR) with expertise in EM education podcasting iteratively revised the items for clarity and relevance. The survey was then pilot tested with 10 residents at the Icahn School of Medicine Emergency Medicine Residency Program at Mt. Sinai to assess for clarity and understanding of the survey questions. No substantial changes were made after pilot testing.

We designed the survey to be completed in less than 10 minutes. Survey completion was voluntary and we provided no compensation for participation. Response rate calculation was based on all non-respondents being eligible, as the survey was sent to specifically named persons who met eligibility requirements. Partially completed surveys were included in response rate.

The final survey consisted of 20 items with questions designed to investigate hypothetical content domains related to listening habits, attention, and motivation (Appendix A). The domain “habits” investigated participants’ setting and activities when listening to podcasts. We aimed to determine the educational environments in which podcasts are being used. The “attention” domain was designed to explore resident attention spans and listening length preferences. The domain “motivation” investigated the reasons why participants choose to listen to EM podcasts. We sought to identify what makes podcasts different than other available educational resources.

### Data Analysis

All data were auto-populated into Google Sheets. We performed statistical analysis in Microsoft Excel (Microsoft Corporation, Redmond, WA). Descriptive statistics were used to evaluate survey data. We reported descriptive statistics in percentages of respondents.

## RESULTS

Of the 605 residents invited to participate, 356 (n= 60.3%) completed the survey. Demographic data are presented in Tables 1 and 2.

### Habits

The mean number of unique EM podcasts that residents subscribe to or regularly listen to was 2.69 (STD 1.89). Two podcasts were the most popular by a wide margin, with 77.8% (n = 277/356) and 62.1% (n = 221/356) regularly listening to *EM:RAP* and the *EMCrit Podcast*, respectively (Appendix B). Most respondents (91.4%, n = 309/356) listen on their smartphones, and about three-quarters (78%, n = 266/356) listen at normal speed (1x). When asked where they find them, 88.7% (n = 300/356) reported they find the podcasts they listen to from word of mouth from other residents, while almost two-thirds (65.7%, n = 222/356) reported finding podcasts based on recommendations from a lecturer or faculty member. The vast majority listen to podcasts at least once a month (88.8%, n = 316/356), and almost half listen at least once a week (48.0%, n = 171/356).

### Attention

**Figure 1:**
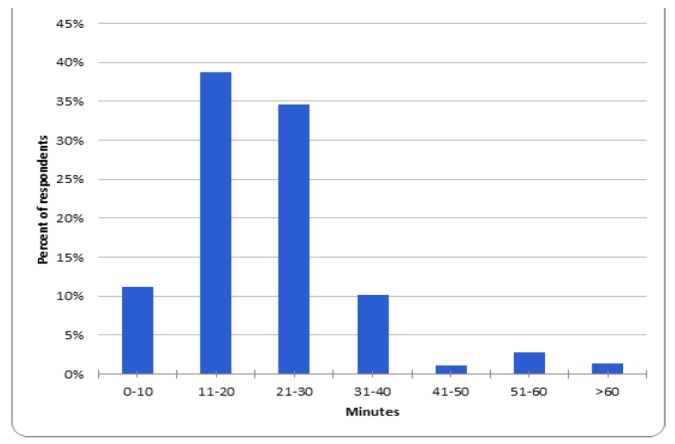
Resident perception of the ideal length of time for an emergency medicine podcast or podcast segment.

When asked what they thought was the ideal length of time for an EM podcast (Figure 1), 38.7% (n = 138/356) answered 11–20 minutes, followed by 21–30 minutes (34.6%, n = 123/356).

When asked if they had ever stopped listening or turned off an EM podcast when they had more time to listen, the top three reasons why they stopped listening were “It was too boring” (57.9%, n = 195/356); “It was not of high quality;” (57.9%, n = 195/356), and “It was too long” (55.2%, n = 186/356).

### Motivation

**Figure 2:**
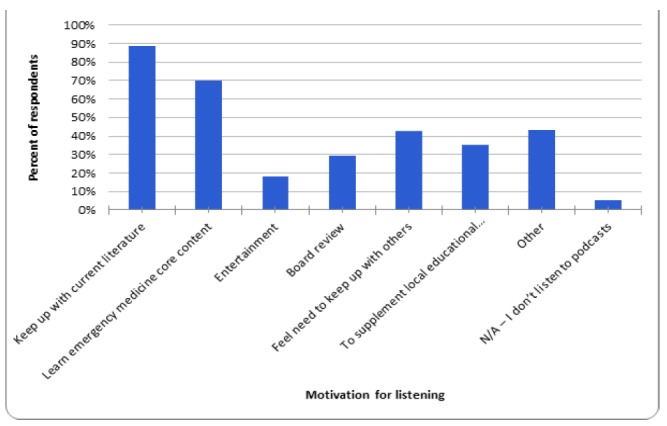
Residents’ motivation for listening to emergency medicine podcasts.

Of those residents who prefer podcasts over other available educational resources (textbooks, blogs, online video, peer-reviewed journals, etc.), they prefer them for their portability (66.9%, n = 238/356), ease of use (66.0%, n = 235/356), and the ability to listen while doing something else (65.5%, n = 233/356). Only 13.8% (n=49/356) said they do not prefer podcasts over other educational resources, while 4.5% (n = 16/356) reported not listening to podcasts. A higher percentage of female respondents (20%, n=24/120) than male respondents (9.8%, n=25/256) said they do not prefer podcasts over other educational resources. Residents reported their motivation to listen to EM podcasts was to “Keep up with current literature” (88.5%, n = 315/356) and “Learn EM core content” (70.2%, n = 250/356), among other answers (Figure 2). Figure 2 details reasons why residents choose to listen to a particular EM podcast.

When asked how much EM podcasts changed their clinical practice, almost three quarters of residents (72.2%, n = 257/356) said podcasts changed their clinical practice either “somewhat” or “very much;” 27.8% (n = 99/356) reported podcasts changed their clinical practice “neutral,” “not much,” or “not at all.”

## DISCUSSION

### Key points

Our data, derived from a diverse cohort of EM residents from across the U.S., suggest that most residents listen to podcasts at least once a month, prefer podcasts less than 30 minutes in length, have several motivations for choosing podcasts, and report that podcasts change their clinical practice.

This work builds on the two recent studies that demonstrated the popularity of asynchronous educational resources among residents by providing a deeper understanding of how and why EM learners are using podcasts.^5^,^6^ The finding that more than 88% of residents listen to podcasts at least every month and the majority listen to two very popular podcasts (*EM:RAP* and *EMCrit*) is consistent with previous studies and highlights the significant influence these two podcasts may be having on resident education.

Resident preference for podcasts less than 30 minutes in length is consistent with national trends in EM toward curriculum delivery in shorter segments.^7^ Podcast creators and EM faculty making curricular decisions may bear this preference in mind. However, no direct evidence exists linking shorter podcasts to better resident retention of information.

Over two-thirds of residents indicated they are motivated to listen to podcasts to learn EM core content. However, the two most popular podcasts that residents listen to (*EM:RAP* and *EMCrit*) are known more for cutting-edge analysis and discussion of controversial new topics than core content. *EM:RAP* has recently re-introduced core content through the C3 Project; however, we did not ask residents to differentiate between main *EM:RAP* content and C3 Project content. The most popular podcasts known to the authors for specifically focusing on core content were regularly listened to by 29.2% (*EM Basic*), and 18.0% (*FOAMcast*) of residents. This may indicate a disconnect between resident expectations of what they’re listening to and what they are actually hearing.

The extent to which podcasts cover the breadth of EM core content is unknown. A recent study of EM online educational resources (OERs) found an imbalanced and incomplete coverage of core content in EM OERs.^18^ Comprehensive and balanced coverage of EM core content is needed if podcasts are going to serve the purposes for which residents are using them. Though several new podcasts have been developed specifically to cover core content topics that may be less represented in other OERs, the balance of core content in podcasts requires investigation.

Further research into the podcast phenomenon should also consider faculty perspectives and experiences. Research into the significance of the gender differences seen in the “motivation” domain will also be important. Qualitative inquiry can provide a deeper understanding of podcasting and may yield a richer theoretical understanding of how and why residents choose podcasts. Finally, a comparison among specific instructional design elements of podcasts may help educators to use podcasts most efficiently.

## LIMITATIONS

Our study has several limitations associated with survey research, chief among them being the small sample size of only 12 residencies. Though we did not see significant variability from those who did not complete the survey, it is possible given our response rate of 60% that our sample is not representative. The Midwest region is underrepresented in our sample and it is not known what impact demographic differences had on the outcomes reported in the study. Despite this, there was also substantial validity evidence inherent to our study design. Some of the survey content was based on published consensus, podcasting experts validated the items’ clarity and relevance, the survey was piloted with a representative group of residents, the study population was well defined, reliable contact information was available for all potential participants, and the response rate was relatively high for a national survey.^5^,^6^,^19^ The use of categorical response options to the survey items was done to increase the response rate. This limits our ability to use parametric statistics to compare groups. While our sampling limits generalizability outside of EM residencies, exclusively studying EM residents allowed specificity to our population of interest.

## CONCLUSION

This survey study informs educators about podcast use among U.S. EM residents. Most residents listen to podcasts at least once a month, prefer podcasts less than 30 minutes in length, have several motivations for choosing podcasts, and report that podcasts change their clinical practice.

## Supplementary Information





## Figures and Tables

**Table 1:** Demographic data of survey respondents, eligible participants, programs involved in the study of educational podcast use, and all allopathic EM programs.

Participant demographics	Respondents (n = 356)	All eligible to participate (n=605)
Age (mean in years)	30.4	Unable to obtain
Gender		
Female	33.4% (n=119)	35.3% (n=164/464)*
Male	65.5% (n=233)	64.7% (n=300/464)*
Decline to state/other	1.1% (n=4)	
Level of training		
PGY-1	27.3% (n=97)	28.1% (n=170/605)
PGY-2	34.0% (n=121)	28.1% (n=170/605)
PGY-3	25.3% (n=90)	28.1% (n=170/605)
PGY-4	13.5% (n=48)	15.7% (n=95/605)

*PGY*, post-graduate year.

* Data obtained from EMRA Match website (https://webapps.acep.org/utils/spa/match#/search/map) on 9/8/2016 of self-reported data from U.S. allopathic EM programs. Twelve of the 182 allopathic programs are dual accredited. None of the programs in the study population are dual accredited. Missing data from study population programs were obtained by contacting faculty at the programs. Missing data from non-study population programs were considered missing and not counted in percentages. The two programs in Puerto Rico were excluded from region calculation.

**Table 2:** Demographic data of programs involved in the study of podcast use, and all allopathic EM programs.

Program demographics	Study programs	All allopathic EM programs
Primary training site		
Military	8% (n=1/12)	5% (8/152)*
Community	17% (n=2/12)	32% (48/152)
University	50% (n=6/12)	54% (82/152)
County	25% (n=3/12)	14% (22/152)
Years of training		
3	50% (n=6/12)	76% (n= 138/182)
4	50% (n=6/12)	24% (n=44/182)
Region		
West	17% (n=2/12)	14% (n=26/180)
Northeast	50% (n=6/12)	30% (n=54/180)
South	25% (n=3/12)	28% (n=51/180)
Midwest	8% (n=1/12)	26% (n=47/180)
ED volume (mean in patients/year)	105,000	89,716

* Data obtained from EMRA Match website (https://webapps.acep.org/utils/spa/match#/search/map) on 9/8/2016 of self-reported data from U.S. allopathic EM programs. Twelve of the 182 allopathic programs are dual accredited. None of the programs in the study population are dual accredited. Missing data from study population programs were obtained by contacting faculty at the programs. Missing data from non-study population programs were considered missing and not counted in percentages. The two programs in Puerto Rico were excluded from region calculation.

* Some military programs also listed as community, university, or county primary training site.

## References

[1] Scott KR, Hsu CH, Johnson NJ (2014). Integration of social media in emergency medicine residency curriculum. Ann Emerg Med.

[2] Cadogan M, Thoma B, Chan TM (2014). Free Open Access Meducation (FOAM): the rise of emergency medicine and critical care blogs and podcasts (2002–2013). Emerg Med J.

[3] EM:RAP Web.

[4] Weingart S Tweet.

[5] Mallin M, Schlein S, Doctor S (2014). A survey of the current utilization of asynchronous education among emergency medicine residents in the United States. Acad Med.

[6] Purdy E, Thoma B, Bednarczyk J (2015). The use of free online educational resources by Canadian emergency medicine residents and program directors. CJEM.

[7] Gottlieb M, Riddell J, Crager SE (2016). Alternatives to the Conference Status Quo: Addressing the Learning Needs of Emergency Medicine Residents. Ann Emerg Med.

[8] Thoma B, Sanders JL, Lin M (2015). The social media index: measuring the impact of emergency medicine and critical care websites. West J Emerg Med.

[9] Mehri M (2015). Factors influencing higher education students to adopt podcast: An empirical study. Computers & Education.

[10] White JS, Sharma N, Boora P (2011). Surgery 101: evaluating the use of podcasting in a general surgery clerkship. Med Teach.

[11] Matava CT, Rosen D, Siu E (2013). eLearning among Canadian anesthesia residents: a survey of podcast use and content needs. BMC Med Educ.

[12] Mostyn A, Jenkinson CM, McCormick D (2013). An exploration of student experiences of using biology podcasts in nursing training. BMC Med Educ.

[13] Mehri M (2015). Factors influencing higher education students to adopt podcast: An empirical study. Computers & Education.

[14] Bandura A (1986). Social foundations of thought and action: A social cognitive theory.

[15] Artino AR, La Rochelle JS, Dezee KJ (2014). Developing questionnaires for educational research: AMEE Guide No. 87. Med Teach.

[16] Phillips AW, Friedman BT, Utrankar A (2017). Surveys of health professions trainees: prevalence, response rates, and predictive factors to guide researchers. Acad Med.

[17] Tuten T (1997). Getting a Foot in the Electronic Door: Understanding Why People Read or Delete Electronic Mail. ZUMA.

[18] Stuntz R, Clontz R (2016). An evaluation of emergency medicine core content covered by free open access medical education resources. Ann Emerg Med.

[19] Barker AL, Wehbe-Janek H, Bhandari NS (2012). A national cross-sectional survey of social networking practices of U.S. anesthesiology residency program directors. J Clin Anesth.

