# Supplementary material for: A Survey of Emergency Medicine Residents’ Use of Educational Podcasts
Source: West J Emerg Med. 2017 Jan 30;18(2):229–34. doi: 10.5811/westjem.2016.12.32850 (PMC5305130; doi:10.5811/westjem.2016.12.32850)
Supplement: Supplementary file 1 [file wjem-18-229-s001.pdf]

Emergency Medicine Resident Podcast Survey

A podcast is a digitally recorded media product that can be downloaded or streamed and is typically an audio file. The questions in this survey refer specifically to emergency medicine podcasts (like EMCrit, EM:RAP, REBEL EM, RAGE, Ultrasound Podcast, Emergency Medicine Case, ER Cast, FOAMcast, etc) and NOT other popular podcasts (Serial, This American Life, etc). Consent is implied by completion of the survey. Your responses are completely anonymous. Thank you for participating.

1. How old are you?
   -dropdown box with integers between 21-50, >50

2. What is your gender?
   A. Female
   B. Male
   C. Decline to state
   D. Other

3. What is your current year of residency training?
   A. Intern
   B. Second Year
   C. Third Year
   D. Fourth Year

4. In what region of the United States do you live?
   A. East Coast
   B. Midwest
   C. South
   D. West Coast
   E. Other

5. How long is your commute to work?
   A. 0-10 Minutes
   B. 11-20 Minutes
   C. 21-30 Minutes
   D. 31-40 Minutes
   E. 41-50 Minutes
   F. 51-60 Minutes
   G. >60 minutes

6. How long is your usual exercise or work-out?
   A. 0-10 Minutes
   B. 11-20 Minutes
   C. 21-30 Minutes

D. 31-40 Minutes
E. 41-50 Minutes
F. 51-60 Minutes
G. >60 minutes

7. How often do you typically listen to emergency medicine podcasts?
A. I don't listen to podcasts
B. Once a year
C. Once a month
D. Once every 2 weeks
E. 1-2 days per Week
F. 3-4 days per Week
G. 5-6 days per Week
H. 7 days per Week

8. How do you find the emergency medicine podcasts you listen to (check all that apply)?
A. Word of mouth from other residents
B. iTunes/Android Search
C. Internet search
D. Recommendation from a lecturer or faculty member
E. Other
F. N/A – I don't listen to podcasts

9. How many unique emergency medicine podcasts programs do you subscribe to or regularly listen to?
A. 0
B. 1
C. 2
D. 3
E. 4
F. 5
G. 6
H. 7
I. 8
J. 9
K. 10
L. >10

10. At what speed do you listen to your podcasts?
A. 1x
B. 1.25x
C. 1.5x
D. 2x
E. >2x
F. Other

G. N/A – I don't listen to podcasts

11. On what device do you listen to your podcasts (check all that apply)?
    A. Desktop Computer
    B. Laptop Computer
    C. Smartphone
    D. MP3/CD player
    E. Other
    F. N/A – I don't listen to podcasts

12. If you had unlimited time, what is the longest time that you would listen to a podcast?
    A. 0-15 minutes
    B. 16-30 minutes
    C. 31-45 minutes
    D. 46-60 minutes
    E. 61-75 minutes
    F. 76-90 minutes
    G. >90 minutes

13. If you have ever stopped listening or turned-off an emergency medicine podcast when you had more time to listen, why did you? (check all that apply)
    A. It was too long
    B. It was too boring/not entertaining
    C. It was not relevant
    D. It was not of high quality
    E. It had conflicts of interest
    F. Other
    G. N/A – I have never stopped listening or turned off a podcast

14. What emergency medicine podcasts do you regularly listen to? (choose all that apply)

Broome Docs
Academic Life in EM Podcast
Annals of Emergency Medicine
CORE EM
Emergency ECG of the Week
Emergency Medicine Cases
Emergency Medicine Ireland
Emergency Ultrasound Podcast
EMCrit
EM Basic
EMJ Club
EM PEM
EM:RAP
Everyday Medicine

ERCast
FOAMcast
Free Emergency Medicine Talks
Hippo EM Resident Call Room
Intensive Care Network
iTeachEM
PEM ED
PEM Playbook
PHARM: Prehospital and Retrieval Medicine
RAGE Podcast
REBEL EM
SMACC Podcast
SMART EM
The Skeptics Guide to Emergency Medicine
The EM Res Podcast
Tox Talk
Other
N/A - I don't listen to podcasts

15. What is the ideal length of time for an emergency medicine podcast or podcast segment?
A. 0-10 Minutes
B. 11-20 Minutes
C. 21-30 Minutes
D. 31-40 Minutes
E. 41-50 Minutes
F. 51-60 Minutes
G. >60 minutes

16. How much do podcasts change your clinical practice?
A. Not at all
B. Not much
C. Neutral
D. Somewhat
E. Very Much

17. If you prefer emergency medicine podcasts over other available educational resources (textbooks, blogs, online video, peer-reviewed journals, etc), why do you prefer podcasts? (check all that apply)
A. Ease of use
B. Portability
C. Entertainment
D. Ability to listen while doing something else
E. Quality of educational content
F. Different perspectives on clinical topics in one resource

G. Current/relevant to your practice
H. Other ____
I. N/A- I do not prefer podcasts over other educational resources
J. N/A – I don't listen to podcasts

18. What is your motivation for listening to emergency medicine podcasts? (check all that apply)
A. Keep up with current literature
B. Learn emergency medicine core content
C. Entertainment
D. Board review
E. Feel need to keep up with others
F. To supplement local educational conferences
G. Other ____
H. N/A – I don't listen to podcasts

19. Why do you choose to listen to a particular emergency medicine podcast? (Check all that apply)
A. The podcast is credible
B. The host/guests have no financial conflict of interest or industry bias
C. It cites its references
D. The content is of good quality
E. The information presented is accurate
F. It is logical, clear, and coherent
G. It is transparent about who is involved in creating the podcast
H. It is entertaining
I. It is current
J. It is composed in a way that is easy to understand
K. It is motivating or inspiring
L. The production quality is professional
M. It clearly differentiates areas at the limits of accepted practice
N. It is possible to leave feedback
O. It is relevant
P. It uses examples, scenarios, and cases to help understand content
Q. It is possible to contact the host or guests
R. Other ____
S. N/A – I don't listen to podcasts

20. Please share any thoughts you have about your experience with emergency medicine podcasts and elaborate on any answers to the above survey questions (we are trying to understand the popularity of podcasts and your perspective is invaluable):

____
____
____
