# Supplementary material for: A Survey of Emergency Medicine Residents’ Use of Educational Podcasts
Source: West J Emerg Med. 2017 Jan 30;18(2):229–34. doi: 10.5811/westjem.2016.12.32850 (PMC5305130; doi:10.5811/westjem.2016.12.32850)
Supplement: Supplementary file 2 [file wjem-18-229-s002.pdf]

Appendix B: What EM podcasts do you regularly listen to? n=356

Podcast	N	%
EM:RAP	277	77.81%
EMCrit	221	62.08%
EM Basic	104	29.21%
ERCast	80	22.47%
FOAMcast	64	17.98%
SMART EM	58	16.29%
REBEL EM	55	15.45%
Academic Life in EM Podcast	43	12.08%
Emergency ECG of the week	42	11.80%
The Skeptics Guide to Emergency Medicine	39	10.96%
Annals of Emergency Medicine	36	10.11%
CORE EM	30	8.43%
SMACC Podcast	24	6.74%
RAGE Podcast	20	5.62%
NA	20	5.62%
EMcast	19	5.34%
emergency ultrasound podcast	19	5.34%
Hippo EM Resident Call Room	19	5.34%
Wildcast EM	15	4.21%
EMMedHome	14	3.93%
Tox Talk	13	3.65%
PEM ED	11	3.09%
Free Emergency Medicine Talks	9	2.53%
Other	8	2.25%

EM PEM	7	1.97%
The EM Res Podcast	7	1.97%
The St. Emlyn's Virtual Hospital Podcast	7	1.97%
HEFT EMCast	6	1.69%
Intensive Care Network	5	1.40%
PEM Playbook	4	1.12%
PHARM: Prehospital and Retrieval Medicine	4	1.12%
EMJ club	3	0.84%
iTeachEM	2	0.56%
Broome Docs	1	0.28%
Emergency medicine cases	0	0.00%
Emergency Medicine Ireland	0	0.00%
Everyday Medicine	0	0.00%
